# Supplementary material for: Impact of drugs and environmental contaminants on amine production by gut bacteria
Source: Mol Syst Biol. 2025 Jun 30;21(10):6. doi: 10.1038/s44320-025-00130-4 (PMC12494721; doi:10.1038/s44320-025-00130-4)
Supplement: Supplementary file 6 — Expanded View Figures [file 44320_2025_130_MOESM6_ESM.pdf]

Expanded View Figures

Figure EV1. Quality control indicators.

(**A**) In each LC-MS run, performance was monitored by regular injection of serially diluted analytical standards, a QC sample (DMSO-control sample from respective bacterial species) and water blanks. All runs were inspected manually and a technical coefficient of variation (CV, indicating the stability of instrument performance) was computed based on the distribution of values obtained for the QC sample. (**B**) Batch variation between each 384-well sample plate was apparent. This was corrected using the strategy described in Methods. In brief, a smoothed line (black) was fitted to outlier-filtered data and the distance of each datapoint to the line was added to the overall median to produce batch-corrected concentration estimates. (**C**) Concentration values after normalisation. (**D**) Hit calling was performed on each sample (ie biological replicate) separately. The deviation of the sample from the fitted line (see B) was divided by the standard deviation of the DMSO controls, for each 96-well culture plate (indicating the degree of combined biological and technical noise in the assay) separately. This z-score was converted to a p value using the normal distribution. (**E**) Distribution of fold changes (relative to mean of DMSO controls) for DMSO controls and xenobiotic-treated samples. As the vast majority of compounds had no effect on a bacterial species, the distributions are similar, although increased density around 0 is observed in treated samples. (**F**) Based on DMSO controls, the baseline median concentration (top) and the overall level of biological and technical noise in the assay (bottom) were quantified. CVs fell between 8 and 18%, a typical range observed in biological mass spectrometry. $N > 600$ biological replicates, error bars indicate the standard deviation. (**G**) Quantile-quantile (Q-Q) plot of fold-change values of DMSO controls across all species and amines. This indicates approximately normal distribution of the values, albeit with slightly heavy tails. (**H**) Power calculation of the Z-test used to determine likelihood that individual datapoints are compatible with the null distribution derived from DMSO controls. The red lines indicate significance thresholds without multiple testing correction ($P < 0.05$) and with Bonferroni correction (1772 compounds). FDR correction with the Benjamini–Hochberg method was applied to the data. The Bonferroni threshold is shown as this is the initial, strictest threshold applied in this method. (**I**) Two biological replicates were recorded for each xenobiotic-species pair. The Pearson correlation between these replicates is indicated in the heatmap. (**J**, **K**) Representative plots illustrating the correlation between the two biological replicates for different metabolite-species pairs.

▶

© The Author(s)

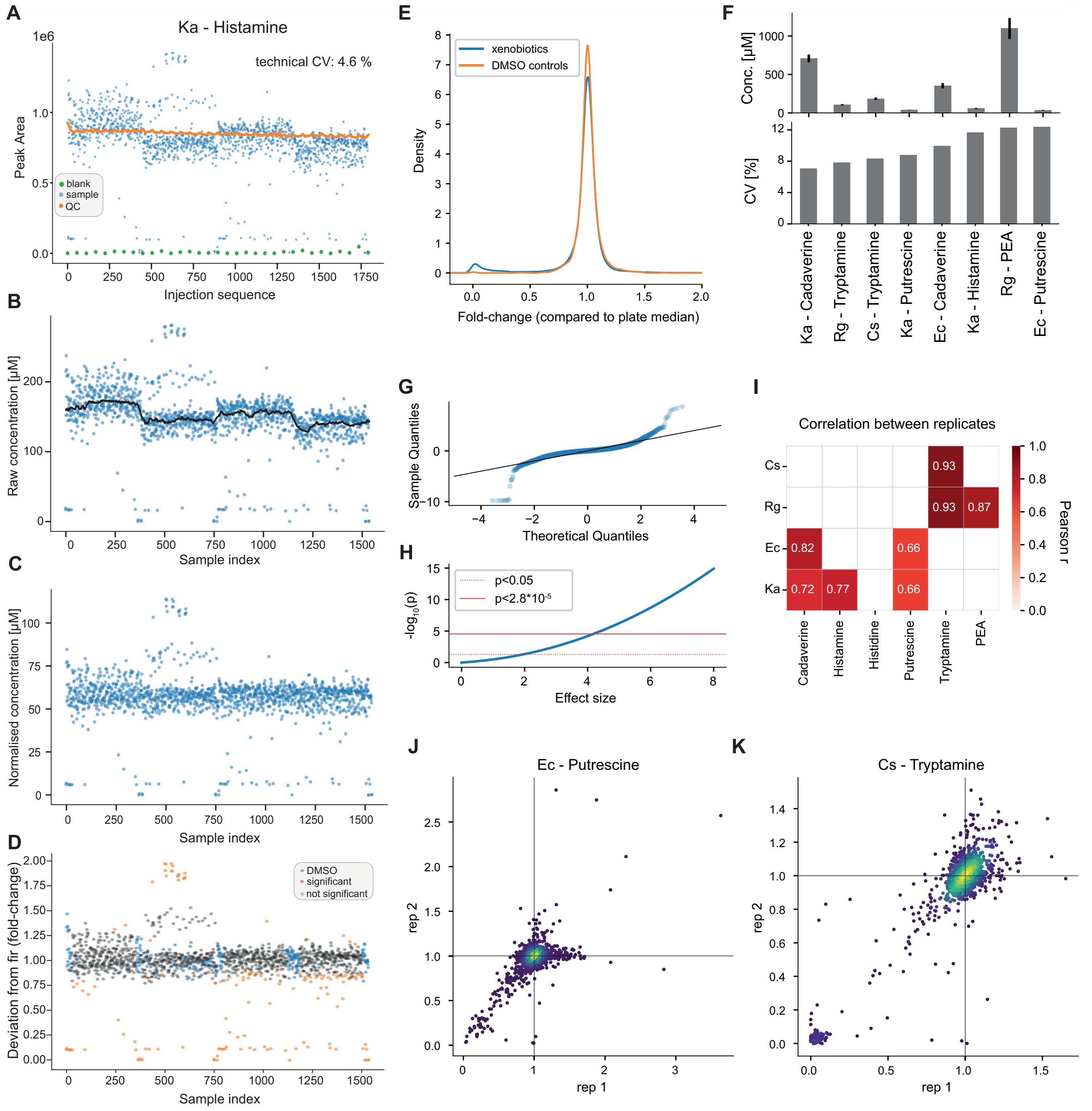
A
Ka - Histamine
1e6
technical CV: 4.6 %
Peak Area
blank
sample
QC
Injection sequence
B
Raw concentration [µM]
Sample index
C
Normalised concentration [µM]
Sample index
D
Deviation from fir (fold-change)
DMSO
significant
not significant
Sample index
E
xenobiotics
DMSO controls
Density
Fold-change (compared to plate median)
F
Conc. [µM]
CV [%]
Ka - Cadaverine
Rg - Tryptamine
Cs - Tryptamine
Ka - Putrescine
Ec - Cadaverine
Ka - Histamine
Rg - PEA
Ec - Putrescine
G
Sample Quantiles
Theoretical Quantiles
H
p<0.05
p<2.8*10-5
-log10(p)
Effect size
I
Correlation between replicates
Cs
Rg
Ec
Ka
0.93
0.93
0.87
0.82
0.66
0.72
0.77
0.66
Cadaverine
Histamine
Histidine
Putrescine
Tryptamine
PEA
Pearson r
J
Ec - Putrescine
rep 2
rep 1
K
Cs - Tryptamine
rep 2
rep 1

 © The Author(s)

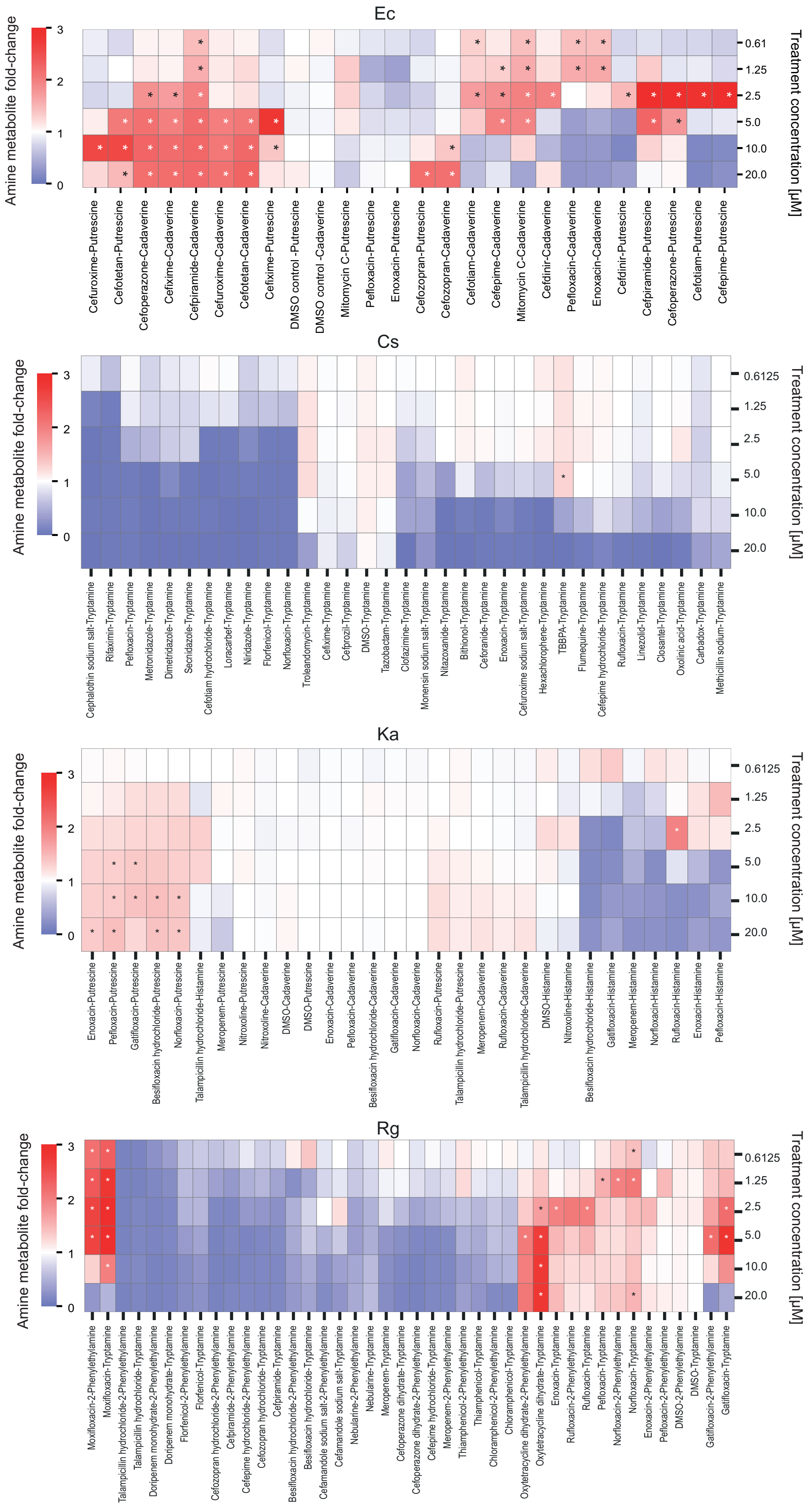
Ec
Amine metabolite fold-change
Treatment concentration [µM]
0.61
1.25
2.5
5.0
10.0
20.0
Cefuroxime-Putrescine
Cefotetan-Putrescine
Cefoperazone-Cadaverine
Cefixime-Cadaverine
Cefpiramide-Cadaverine
Cefuroxime-Cadaverine
Cefotetan-Cadaverine
Cefixime-Putrescine
DMSO control -Putrescine
DMSO control -Cadaverine
Mitomycin C-Putrescine
Pefloxacin-Putrescine
Enoxacin-Putrescine
Cefozopran-Putrescine
Cefozopran-Cadaverine
Cefotiam-Cadaverine
Cefepime-Cadaverine
Mitomycin C-Cadaverine
Cefdinir-Cadaverine
Pefloxacin-Cadaverine
Enoxacin-Cadaverine
Cefdinir-Putrescine
Cefpiramide-Putrescine
Cefoperazone-Putrescine
Cefotiam-Putrescine
Cefepime-Putrescine
Cs
0.6125
Cephalothin sodium salt-Tryptamine
Rifaximin-Tryptamine
Pefloxacin-Tryptamine
Metronidazole-Tryptamine
Dimetridazole-Tryptamine
Secnidazole-Tryptamine
Cefotiam hydrochloride-Tryptamine
Loracarbef-Tryptamine
Niridazole-Tryptamine
Florfenicol-Tryptamine
Norfloxacin-Tryptamine
Troleandomycin-Tryptamine
Cefixime-Tryptamine
Cefprozil-Tryptamine
DMSO-Tryptamine
Tazobactam-Tryptamine
Clofazimine-Tryptamine
Monensin sodium salt-Tryptamine
Nitazoxanide-Tryptamine
Bithionol-Tryptamine
Ceforanide-Tryptamine
Enoxacin-Tryptamine
Cefuroxime sodium salt-Tryptamine
Hexachlorophene-Tryptamine
TBBPA-Tryptamine
Flumequine-Tryptamine
Cefepime hydrochloride-Tryptamine
Rufloxacin-Tryptamine
Linezolid-Tryptamine
Closantel-Tryptamine
Oxolinic acid-Tryptamine
Carbadox-Tryptamine
Methicillin sodium-Tryptamine
Ka
Enoxacin-Putrescine
Pefloxacin-Putrescine
Gatifloxacin-Putrescine
Besifloxacin hydrochloride-Putrescine
Norfloxacin-Putrescine
Talampicillin hydrochloride-Histamine
Meropenem-Putrescine
Nitroxoline-Putrescine
Nitroxoline-Cadaverine
DMSO-Cadaverine
DMSO-Putrescine
Enoxacin-Cadaverine
Pefloxacin-Cadaverine
Besifloxacin hydrochloride-Cadaverine
Gatifloxacin-Cadaverine
Norfloxacin-Cadaverine
Rufloxacin-Putrescine
Talampicillin hydrochloride-Putrescine
Meropenem-Cadaverine
Rufloxacin-Cadaverine
Talampicillin hydrochloride-Cadaverine
DMSO-Histamine
Nitroxoline-Histamine
Besifloxacin hydrochloride-Histamine
Gatifloxacin-Histamine
Meropenem-Histamine
Norfloxacin-Histamine
Rufloxacin-Histamine
Enoxacin-Histamine
Pefloxacin-Histamine
Rg
Moxifloxacin-2-Phenylethylamine
Moxifloxacin-Tryptamine
Talampicillin hydrochloride-2-Phenylethylamine
Talampicillin hydrochloride-Tryptamine
Doripenem monohydrate-2-Phenylethylamine
Doripenem monohydrate-Tryptamine
Florfenicol-2-Phenylethylamine
Florfenicol-Tryptamine
Cefozopran hydrochloride-2-Phenylethylamine
Cefpiramide-2-Phenylethylamine
Cefepime hydrochloride-2-Phenylethylamine
Cefozopran hydrochloride-Tryptamine
Cefpiramide-Tryptamine
Besifloxacin hydrochloride-2-Phenylethylamine
Besifloxacin hydrochloride-Tryptamine
Cefamandole sodium salt-2-Phenylethylamine
Cefamandole sodium salt-Tryptamine
Nebularine-2-Phenylethylamine
Nebularine-Tryptamine
Meropenem-Tryptamine
Cefoperazone dihydrate-Tryptamine
Cefoperazone dihydrate-2-Phenylethylamine
Cefepime hydrochloride-Tryptamine
Meropenem-2-Phenylethylamine
Thiamphenicol-2-Phenylethylamine
Thiamphenicol-Tryptamine
Chloramphenicol-2-Phenylethylamine
Chloramphenicol-Tryptamine
Oxytetracycline dihydrate-2-Phenylethylamine
Oxytetracycline dihydrate-Tryptamine
Enoxacin-Tryptamine
Rufloxacin-2-Phenylethylamine
Rufloxacin-Tryptamine
Pefloxacin-Tryptamine
Norfloxacin-2-Phenylethylamine
Norfloxacin-Tryptamine
Enoxacin-2-Phenylethylamine
Pefloxacin-2-Phenylethylamine
DMSO-2-Phenylethylamine
DMSO-Tryptamine
Gatifloxacin-2-Phenylethylamine
Gatifloxacin-Tryptamine

◀ **Figure EV2. Dose–response behaviour for selected xenobiotics.**

Heatmaps show amine metabolite fold changes across species (panels), compound-metabolite pairs (rows) and treatment concentrations (columns). Hits (same criteria as main screen: mean $\log_2$(fold change) >0.32, both replicates $P_{adj} < 0.05$ (two-sided FDR-corrected z-test), consistent direction of metabolite change) with increased amine production are marked with '*'. Heatmaps columns were clustered using Euclidean distance and the Average method.

 © The Author(s)

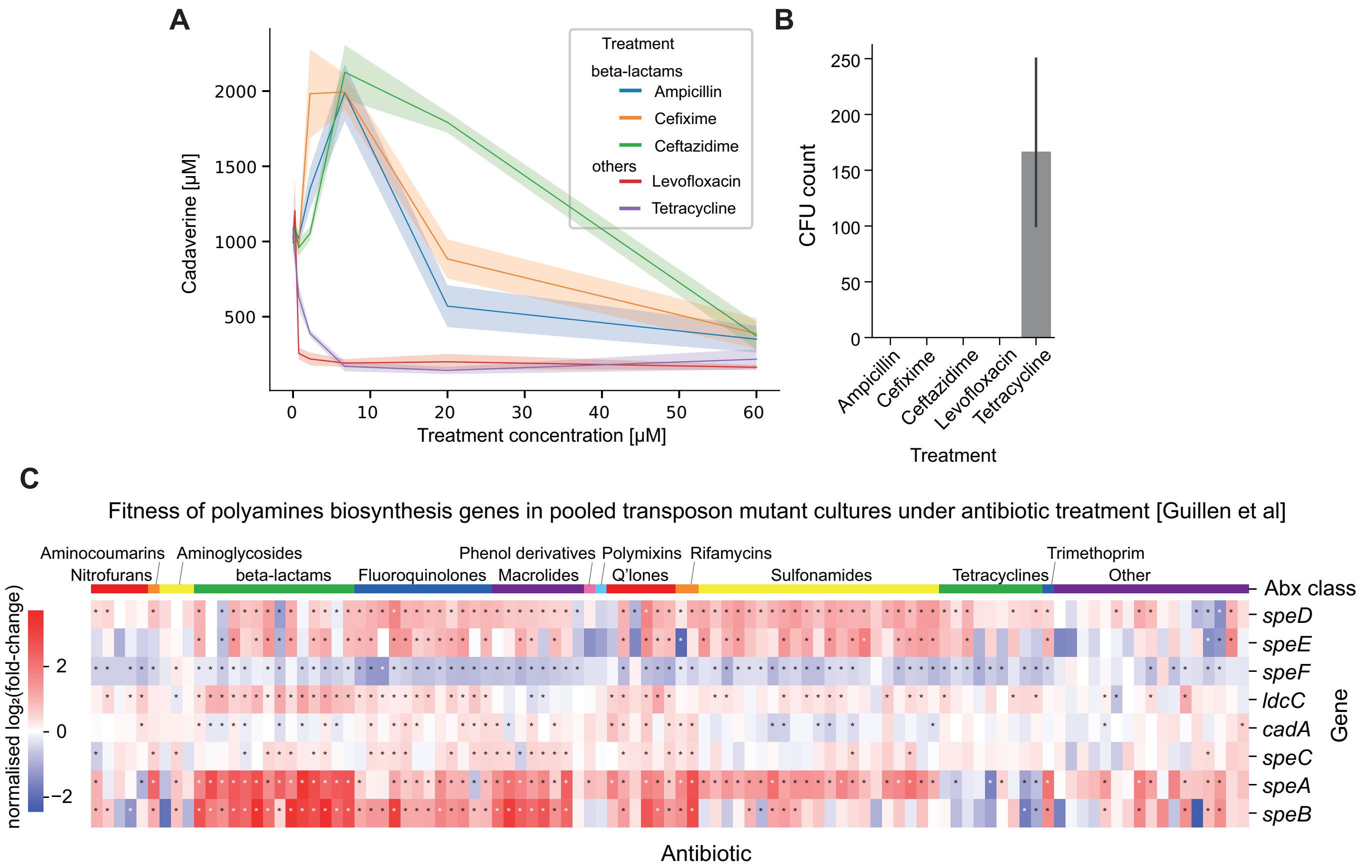

Figure EV3. Additional antibiotics experiments.

(**A**) Validation of the main screen results using a subset of antibiotics from different classes, prepared and measured independently. Lines indicate the mean and shaded areas the standard deviation of $n = 3$ biological replicates. (**B**) Number of colony-forming units of *E. coli* after 3.5 h incubation in mGAM medium with 20 µM of each antibiotic. Bar heights indicate the mean and error bars the standard deviation of $n = 3$ biological replicates. (**C**) Analysis of published dataset by Guillen et al (Noto Guillen et al, 2024) capturing the fitness of pooled, genome-wide knock-out mutants in the presence of various drugs. The analysis is based on Supplementary Table 3 in the above publication, using the normalised log_2(fold-change) data (sheet 'normLFC') and adjusted *P* values (sheet 'P_{adj}', see the source publication for details). We filtered the data for known polyamine biosynthetic enzymes (*y* axis) and antibiotics (column antibiotic == 1). Significant interactions are marked '*', using the same cut-off of $P_{adj} < 0.25$ as used by the authors. Rows were clustered using Euclidean distance and the Average method. Columns were sorted by antibiotic class.

© The Author(s)

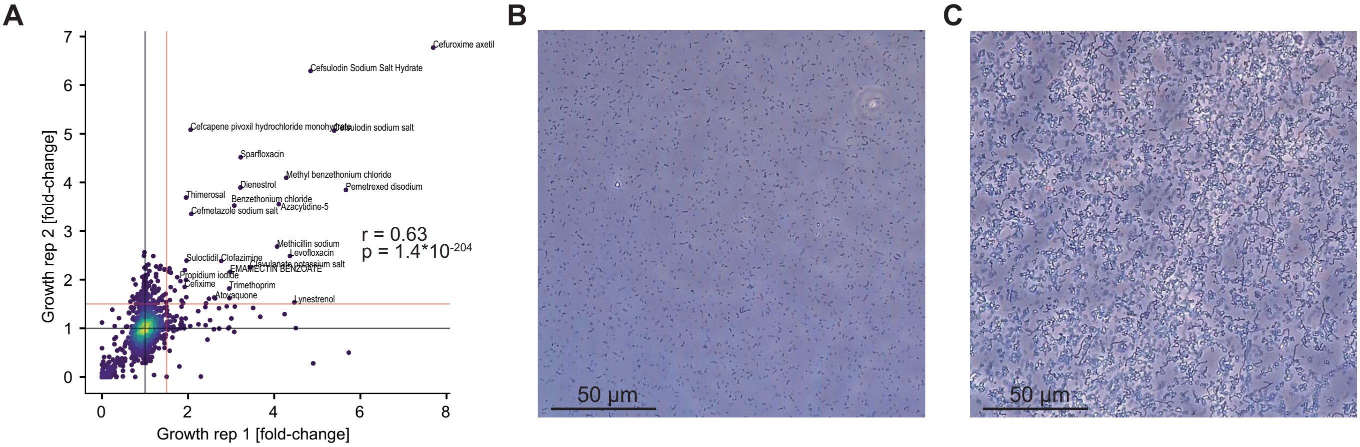

Figure EV4. Cell aggregation in *R. gnavus* confounds OD measurements.

(**A**) Growth fold changes for *R. gnavus* across the two biological replicates. The Pearson correlation coefficient and associated two-sided *P* value is shown in the plot. Very high values are often consistent across replicates. (**B**) Phase contrast micrograph of *R. gnavus* cells in control conditions (mGAM 1% DMSO). Images were acquired using a Leica DM1000 LED microscope, a 20x/0.4 PH1 objective and Leica ICC50 W camera. (**C**) Similar micrograph of *R. gnavus* cells treated with compound causing abnormally high OD readings.

 © The Author(s)
